# Scirrhous colon cancer presenting as a pseudokidney sign

**DOI:** 10.1002/ccr3.3198

**Published:** 2020-08-05

**Authors:** Takayuki Yamada, Susumu Ohwada

**Affiliations:** ^1^ Director Asunaro Clinic Takasaki Gunma Japan; ^2^ Director ASKOHWADA Consultation Clinic of Gastroenterology and Oncology Maebashi Gunma Japan

**Keywords:** colon cancer, pseudokidney sign, scirrhous

## Abstract

This case presents ascending colon cancer with a typical pseudokidney sign on ultrasonography. The cancer extended from the ascending colon to the terminal ileum without intussusception. The pseudokidney sign may represent colon scirrhous cancer.

## Graphical abstract

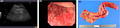

A 35‐year‐old man having diarrhea and right lower abdominal pain showed a pseudokidney sign, indicating right‐sided colon cancer with intussusception on ultrasonography. Colonoscopy showed circumferential stenosis with 30‐cm long scirrhous carcinoma from the ascending colon to terminal ileum. We report on colon scirrhous cancer and considering pseudokidney sign as cancer‐bearing.

A 35‐year‐old man visited us for prolonged diarrhea and right lower abdominal pain lasting >1 week. Physical examination revealed a baseball‐sized tender tumor. Ultrasonography detected a hypoechoic portion with a central hyperechoic region, confirming a typical pseudokidney sign^1^, ^2^ ^,^ (Figure 1A), indicating right‐sided colon cancer with intussusception. Colonoscopy showed circumferential long ascending colon stenosis with cobblestones nodularity (Figure 1B). Biopsy examination revealed poorly differentiated adenocarcinoma. Computed tomography showed extensive metastases to the mesenteric, mediastinal, and left supraclavicular lymph nodes. Stage Ⅳ scirrhous colon cancer was diagnosed. He underwent open right hemicolectomy for colonic obstruction. The tumor was a 30‐cm long scirrhous carcinoma extending till the ascending colon, Bauhin's valve, appendix, and terminal ileum without intussusception (Figure 1C). He received adjuvant chemotherapy but died of cancer 8 months postoperatively.

**FIGURE 1:**
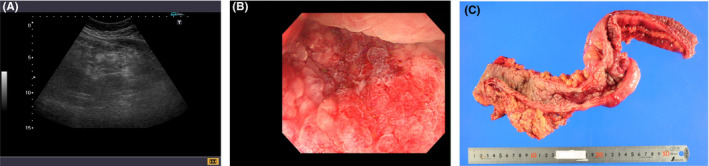
A, Ultrasonography detecting a hypoechoic portion with a central hyperechoic region, confirming a pseudokidney sign. B, Colonoscopy showing circumferential long ascending colon stenosis with cobblestones nodularity. C, The tumor extending till the ascending colon, Bauhin's valve, appendix, and terminal ileum without intussusception

This report describes the scirrhous spread of colon cancer and emphasizes the need for physicians to recognize the pseudokidney sign as cancer‐bearing.

## CONFLICT OF INTEREST

None declared.

## AUTHOR CONTRIBUTIONS

TY: served as a diagnostician and first author; SO: served as a supervisory doctor.
